# Tissue Expression of Programmed Cell Death 1 Ligand1 (PD-L1) in Biopsies of Transplant Livers of Pediatric Patients as a Possible Marker of Acute Cellular Rejection

**DOI:** 10.3390/jcm12134269

**Published:** 2023-06-26

**Authors:** Sylwia Szymanska, Malgorzata Markiewicz-Kijewska, Michal Pyzlak, Agnieszka Karkucinska-Wienckowska, Mateusz Ciopinski, Piotr Czubkowski, Piotr Kaliciński

**Affiliations:** 1 Department of Pathology, The Children’s Memorial Health Institute, 04-736 Warsaw, Poland; 2 Department of Pediatric Surgery and Organ’s Transplantation, The Children’s Memorial Health Institute, 04-736 Warsaw, Poland; 3 Warsaw Department of Pathology, The Institute of Mather and Child, 01-211 Warsaw, Poland; mpyzla@patomorfologia.com; 4 Department of Gastroenterology, Hepatology, Nutrition Disorder and Pediatric, The Children’s Memorial Health Institute, 04-736 Warsaw, Poland

**Keywords:** pediatric liver transplantation, liver biopsy, PD-L1, ACR, immune tolerance

## Abstract

Introduction: Preclinical models have demonstrated that PD-1 and its ligand programmed death ligand1 (PD-L1) play significant roles in both graft induction and the maintenance of immune tolerance. It has also been suggested that PD-L1 tissue expression may predict graft rejection; however, the available data are sparse and inconclusive. Some studies were conducted on patients with cancer; most of them do not concern the liver, especially within the context of the use of immunohistochemical tests. Therefore, the aim of our study was to assess the relationship between tissue expression of PD-L1 in a unique material, i.e., in the liver biopsies of pediatric patients after transplantation with the presence of acute cellular rejection (ACR). Material and Methods: This retrospective study enrolled 55 biopsies from 55 patients who underwent protocol liver biopsies. The control group consisted of 19 biopsies from 13 patients diagnosed with acute cellular rejection (rejection activity index/RAI/ from 2 to 8). An immunohistochemical (IHC) staining for PD-L1 was performed in all of the liver specimens; its expression was analyzed in different regions of liver tissue (in inflammatory infiltrates and within the endothelium and hepatocytes). The following changes were re-evaluated in each specimen: features of any kind of rejection (acute cellular, antibody-mediated, chronic); the presence and severity of fibrosis (Ishak scale); and the presence of cholestasis and steatosis. Clinical parameters were also evaluated, including tests of liver function (AST, ALT, GGT, bilirubin). Results: The age of patients in the study group ranged from 2.37 to 18.9 years (median 13.87 years), with the time after transplantation being 1–17 years (median 8.36 years). The age of patients in the control group ranged from 1.48 to 17.51 years (median 7.93 years), with their biopsies being taken 0.62–14.39 years (median 1.33 years) after transplantation. We found a statistically significant relationship between PD-L1 expression on inflammatory infiltrates and ACR; however, there was no statistically significant relationship between PD-L1 endothelial expression and ACR. PD-L1 was not positive in the hepatocytes regardless of if it was the study or control group that was under observation. Conclusion: PD-L1 appears to be a promising marker to predict graft rejection.

## 1. Introduction

The transplantation of solid organs, such as livers, kidneys, and hearts, is currently recognized as the most effective method of treating the end-stage failure of these organs. The quality of life of transplant patients is almost the same as before surgery. Unfortunately, we are still unable to fully control the immune processes that lead to the loss of grafts. New antibodies, fusion proteins, and low-molecular weight drugs are constantly being researched. The use of immune checkpoint inhibitors in solid organ transplant recipients has recently been widely investigated [1,2,3,4]. Among these inhibitors, the programmed-death 1 (PD1) receptor and its ligands PD-L1 (B7-H1) and PD-L2 (B7-DC) have been well characterized. PD1 is a transmembrane glycoprotein that belongs to the CD28/B7 family and is encoded in humans by the pdcd1 gene, whose locus is 2q37.3. It is induced on CD4 and CD8 T cells, B cells, NK cells, monocytes, and activated dendritic cells [5,6]. Moreover, it is constitutively expressed by a variety of parenchymal cells, including in the heart, lung, kidney, pancreas, and liver. PD-L1 and its ligands inhibit the signal that is transmitted from activated T lymphocytes, and they also reduce the expression of pro-inflammatory cytokines and anti-apoptotic molecules. All of these phenomena lead to the inhibition of the activation of the immune system, which enables lymphocyte immune tolerance in relation to, among others, transplanted organs [7,8,9,10]. In the liver, PD-L1 is expressed by sinusoidal endothelial cells (LSECs), Kupffer cells (KC), stellate cells, and hepatocytes [11]. Although PD-L1 status has been mainly investigated in the context of tumors (especially non-small cell lung cancer), it has also been proposed as a potential new tool for predicting rejection risk in several studies [12,13,14,15]. However, so far, the number of patients evaluated was small, and the impact of these findings should be confirmed in larger group cases. Most papers have focused on the heart and kidneys, whereas some have only been carried out on mouse models. Only two available studies [1,16] have focused on patients after liver transplantation and PD-L1 expression. Antibodies against PD-L1 that can be used in tissue biopsies or surgical materials are commercially available. Core-needle biopsy, referring to both the protocol one and the one performed to diagnose lesions in patients with clinical symptoms/laboratory test abnormalities, are routinely performed in liver recipients; thus, the use of immunohistochemical tests (IHCs) seems to be a quick and relatively inexpensive way to monitor different processes in this organ. Nevertheless, histopathological changes are sometimes difficult to interpret and require close correlation with clinical data. Thus, we are constantly looking for new predictive and diagnostic markers that will help to optimize and improve the diagnosis of patients after transplantation. Moreover, different targeted therapies are now available, so the in vivo determination of various markers may be helpful for making decisions about further treatment. Based on all of these facts, the primary aim of our study was to assess the relationship between liver tissue expression of PD-L1 with the presence of acute cellular rejection (ACR) and other graft damage (thorough the reassessment of all biopsies that had been performed). We also focused on the precise histopathological analysis of specimens so that the secondary endpoints included the assessment of PD-L1 expression in different cells, not only in lymphocytes (inflammatory infiltration) but also within endothelial cells and hepatocytes. Finally, in order to verify the potential role of PD-L1 as a predictive marker, we compared the obtained results with the results of basic laboratory tests from the last available follow-up visits with our patients. Thus, our work is so far the most holistic approach to the subject among the data in the literature.

## 2. Material and Methods

### 2.1. Study Design

At the Children’s Memorial Health Institute, protocol biopsies from patients after liver transplantation have been taken since 2016. They are taken 1, 5, and 10 years after transplantation. Patients enrolled in this single-center pilot study had liver transplantations performed between June 2000 and January 2010. The primary objective was to assess the relationship between liver tissue expression of PD-L1 with the presence of acute cellular rejection (ACR) and graft damage. The secondary endpoints included the assessment of PD-L1 in different cells and liver compartments as well as the determination of whether this expression has any significant meaning.

We performed a retrospective analysis of 74 biopsies specimens that were taken from 68 pediatric patients. In the study group, we involved 55 patients who underwent 55 protocol liver biopsies. In the control group, we enrolled 13 patients who had 19 liver biopsies due to acute rejection.

### 2.2. Histopathology

All tissue samples were fixed in 4% formalin and embedded in paraffin. The paraffin 4 um sections were routinely stained with hematoxylin and eosin (H&E). PD-L1 (rabbit monoclonal primary antibody VENTANA (SP142)) was performed in each biopsy according to the manufacturer’s instructions. Two experienced pathologists were involved in independently determining the immunostaining signals. The pathologists knew when the patient had undergone the transplantation and that it was a protocol liver biopsy. The proportion of inflammatory cells (lymphocytes and macrophages) as well as endothelial cells or hepatocytes in each chosen field was determined by conducting a manual count of individual cells using a × 10 objective lens. Only unequivocal membranous staining that was recognizable using a × 10 objective lens was regarded as a positive finding. The positive staining cutoff point was set at ≥ 1% based on a previous available study [1]. The determinations regarding the positivity of a staining were performed based on a consensus between the two pathologists. Additionally, C4d (Biomedica Group, dilution 1:40) was used as a marker of antibody-mediated rejection (AMR). Histopathological changes such as features of cellular and/or humoral rejection, chronic rejection, presence and severity of fibrosis (Ishak scale), and presence of cholestasis and steatosis were also evaluated. The severity of ACR was assessed using the rejection activity index (RAI) [17].

### 2.3. Statistic Analysis

The data were analyzed using Statistica 13.3 software (StatSoft Polska, 30-110 Kraków, Poland). Due to a significant difference in the size of the groups and no normal distribution of the characteristics of the subjects, the data were analyzed using the Mann–Whitney U test. Differences were considered statistically significant when *p*-values were less than 0.05 (*p* < 0.05).

The following data were compared:

- PD-L1 expression in endothelium, inflammatory infiltrates, and hepatocytes in both groups;
- Severity of fibrosis in relation to PD-L1 expression in endothelial and inflammatory infiltrates;
- Laboratory test results (GGT, AST, ALT, bilirubin) at the time of biopsy against to the last available results taken at the time of the follow-up visit.

## 3. Results

### Clinical Features

In the study group, we involved 55 patients after liver transplantation, who were aged 2.37–18.9 years (median 13.87 years) who underwent 55 protocol liver biopsies, which were taken 1–17 years after liver transplantation (median 8.36 years). Children were transplanted within the ages of 0.12–17.9 years (mean 5.13 years, median 2.97 years). Most of the children were transplanted due to biliary atresia (32 patients 58%). Four patients underwent retransplantation. Thirty-three patients were electively transplanted, and nineteen patients were transplanted urgently or due to the decompensation process of chronic liver disease. Thirty-six children were transplanted using grafts from a living related donor. 

In the control group, we enrolled 13 patients who were aged 1.48–17.51 years (median 7.93 years) and who had 19 liver biopsies due to acute rejection. Liver biopsies were taken in the control group 0.62–14.39 years (median 1.33 years) after transplantation. Five children underwent transplantation due to biliary atresia, and four patients had retransplantation. One patient received two liver grafts and had acute rejection episodes after each transplantation. Nine children received grafts from living related donors.

In the protocol biopsies group, none of the patients had AMR diagnosed in biopsy. In 21 of the patients, the pathomorphological examination did not show any significant deviations (biopsy within normal limits). Steatosis of a mild (up to 30% of the core) to moderate (up to 60% of the core) degree was found in four of the patients. Significant cholestasis was visible in seven biopsies, and minimal was observable in another five. The most common changes were nonspecific inflammatory infiltrates (described in 17 biopsies) of varying severity not meeting the ACR criteria. In most cases, there were discrete chronic inflammatory infiltrates; in one biopsy, the lesions had average activity, whereas they formed into lymph nodules in two other biopsies. Recurrence of primary sclerosing cholangitis (PSC), which is defined as the presence of “onion skin” fibrosis around the affected bile ducts, was observed in one patient.

The ACR group consisted of 19 biopsies from 13 patients diagnosed with acute cellular rejection. The RAI score ranged between two and eight (Table 1). In one liver biopsy, we found mild steatosis. In six biopsies, we found significant cholestasis, and in another one, we observed minimal cholestasis.

Fibrosis was assessed on a six-point Ishak scale. The detailed distribution of the results of both groups is presented below (Table 2).

**Figure 1:**
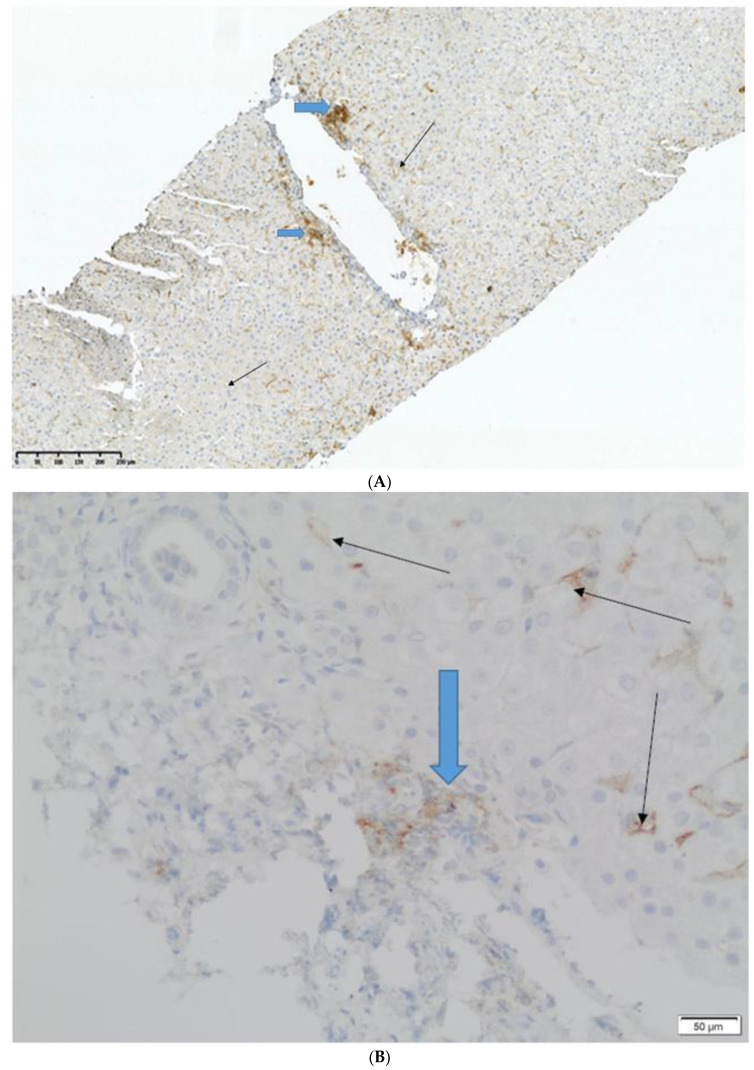
Images showing the tissue expression of PD-L1. Black arrows are the expression in endothelial cells and blue arrows are the expression in inflammatory infiltrates. (**A**) Magnification ×40, (**B**) Magnification ×100.

There was no chronic rejection (ductopenia) or AMR incident in any biopsy (C4d stained negative in all biopsies, no portal microvascular endothelial cell enlargement). PD-L1, if present, was positive either on allograft lymphocytes or endothelial cells in both groups (Figure 1A,B); it was not positive in the hepatocytes, regardless of if we were examining the study or control group.

The positive staining (> 1%) of PD-L1 in the endothelium was detected in 13 out of 19 (68%) ACR biopsies, while in the inflammatory infiltrates, staining was detected in 4 out of 19 (21%) ACR biopsies. In the protocol biopsies, 44 out of 73 (60%) had expression in the endothelium and 4 out of 73 (5.5%) had expression in the inflammatory infiltrates.

**Figure 2:**
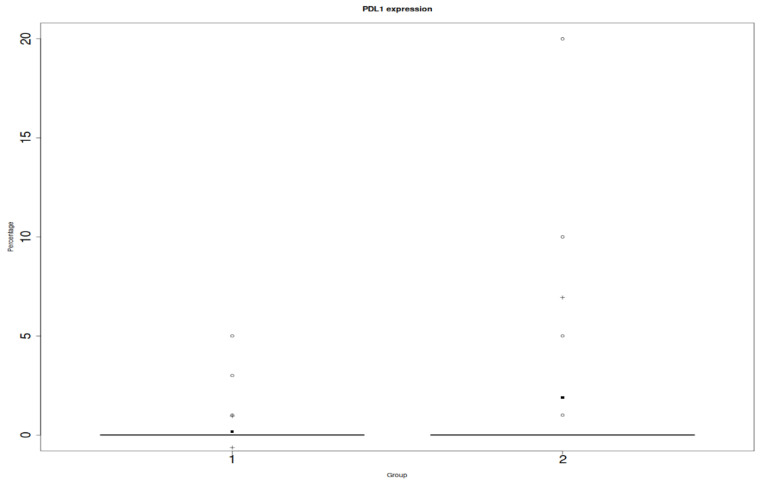
Y: percentage of positive cells in in the entire biopsy; x: PD-L1 on inflammatory infiltrates; 1: protocol biopsies; 2: ACR biopsies. Differences were considered statistically significant when *p*-values were less than 0.05 (*p* < 0.05).

**Figure 3:**
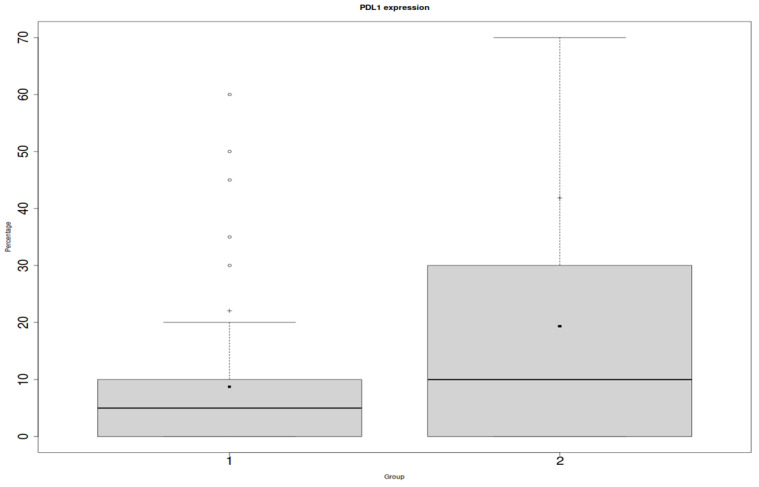
Y: percentage of positive cells in the entire biopsy; x: PD-L1 in endothelial cells; 1: protocol biopsies; 2: ACR biopsies.

We found a statistically significant relationship between PD-L1 on the inflammatory infiltrates and ACR (*p* = 0.04), which is shown in Figure 2. However, we did not prove a relationship between PD-L1 endothelial expression and the ACR (*p* = 0.15) (Figure 3).

Differences were considered statistically significant when *p*-values were less than 0.05 (*p* < 0.05). The clinical characteristics of the patients and treatment are presented below (Table 3, Table 4 and Table 5).

There was no significant relationship between PD-L1 expression and the severity of fibrosis at any location, as the *p*-values were *p* = 0.1251 for endothelial expression and *p* = 0.1329 for inflammatory infiltrates. A statistical analysis of the most recent enzymes level (bilirubin, AST, ALT, GGT) in comparison to the last available results was also performed. The only significance in the differences was found in the bilirubin levels (*p* = 0.04).

## 4. Discussion

PD-L1, which is a transmembrane protein that is involved in immune modulation, serves as a checkpoint inhibitor. Regulation of its expression and functionality is a complex network involving different cytokines and molecules with varying relevance in the individual modulators in different cell types [18]. PD-L1 status has been widely investigated in tumors as the agents of enhancing the tumor-specific activity of immune cells. Recently, its role in immune tolerance in transplant organs has also been analyzed. For instance, PD-L1 has been shown to be critical for the maintenance of peripheral tolerance in the context of transplantation, as the blockage of the pathway was shown to lead to a fastened graft rejection in CD28 and B7-1/B7-2 double-deficient models [5,6]. It is also believed that the tissue expression of PD-L1 in a donor organ is necessary to prevent chronic allograft rejection and other in situ graft diseases. Kaul et al. [19] indicated that the increased tissue expression of PD-L1 in the transplanted heart is associated with a faster descent of the process of acute cellular rejection. On the other hand, Lipson et al. [20] presented a case report of A 57-year-old woman who underwent kidney transplantation and developed cutaneous squamous-cell carcinoma as a result of the applied therapy. As a result, she was administered anti–PD-1 drugs, but unfortunately had an ACR and finally lost her graft. Therefore, it is important to remember that PD-L1 may play different roles where immunogenic tolerance is concerned, and its roles depend on its location. Moreover, anti-PD-1 drugs do not possess intrinsic cytotoxicity and have a lack of adverse events observed due to their mechanism of action; therefore, they could potentially be effective in transplant patients [21]. The fear of their use is the possibility of patients developing ACR, as PD-L1 elevated levels have been associated with cellular rejection in several studies [12,13,14,15]. Unfortunately, the studies on these subjects are scarce and ambiguous. Thus, we decided to assess the relationship between liver tissue expression of PD-L1 with ACR and graft damage. We were the first to determine PD-L1 expression in different cells and liver tissue compartments. We enrolled 55 patients in this retrospective analysis which, to the best of our knowledge, is the largest investigated group so far. Our outcomes are consistent with DeLeon et al. [1], who studied PD-L1 tissue expression in liver transplant recipients and suggested its relationship with ACR; however, their group consisted of only five cases. The authors found that in three patients without allograft rejection, 0% of PD-L1 staining was observed, whereas both cases of ACR in this cohort were found to have allograft lymphocyte PD-L1 expression with a median PD-L1 lymphocyte expression of 27.5%. In our study, in the protocol biopsies with small inflammatory infiltrates, PD-L1 expression was 0% in most cases and 1% in one case. Meanwhile, in the ACR biopsies, positive expression was up to 20%. Unfortunately, apart from the presence of rejection, not much is known about other histopathological lesions in the analyzed biopsies. Moreover, in contrast to our group, the patients studied by the above-mentioned study group received treatment with PD-L1 inhibitors, and the main assumption of the study was to assess the effect of drugs on the likelihood of developing a rejection. The authors themselves emphasized that their pilot study required confirmation on a larger group of patients. Friend et al. [22] also suggested a link between PD-L1 and ACR, which is consistent with our observations. The authors reported two cases of patients who developed ACR and had elevated PD-L1 expression. In this study, the marker levels were determined using immunofluorescence. Despite the availability of biopsies, the authors did not detect PD-L1 in the tissue. Unfortunately, due to the retrospective character of our study, we could not compare PD-L1 tissue expression with immunofluorescence. However, not all hospitals have the ability to perform immunofluorescence tests, whereas IHC tests are available in almost every department of pathomorphology. Thus, the assessment of PD-L1 tissue expression appears to be more practical. Nonetheless, our pilot study may be an introduction for conducting a prospective study on a larger number of patients while comparing different methods of PD-L1 detection. Although PD-L1 is expressed by sinusoidal endothelial cells (LSECs), Kupffer cells (KC), stellate cells, and hepatocytes, data on the relation between PD-L1 and liver transplants are limited and basic. In our study, we decided to evaluate its presence in different cells and compartments of the organ. Generally speaking, the innovation of our work is a precise histopathological evaluation of the liver specimens, while other papers have mostly concentrated on the presence of a rejection. According to the available literature data, statistically significant differences in PD-L1 expression were obtained in the inflammatory infiltrates. Nevertheless, the percentage of positively staining cells in both groups was higher when they were expressed in the vascular epithelium. Surprisingly, the hepatocytes did not show a positive reaction at all regardless of the groups. This finding is very promising for the differentiation of ACR and other non-specific inflammatory infiltrates, especially in equivocal cases. PD-L1 may be useful not only as a prognostic but also as a diagnostic marker, especially now that more and more patients are candidates for liver transplantation after receiving immunotherapy to downstage hepatocellular carcinoma [22,23]. In these patients, rejection is a major concern; thus, prompt and correct diagnosis is essential in these cases. We also found a dependency between the bilirubin levels in the most recent tests available in our patients. It may support the thesis by Portuguese et al. [12] that patients with positive allograft PD-L1 staining may be candidates for closer monitoring due to a higher risk of further complications and liver damage; our observations, however, are not strong enough to draw firm conclusions. It is certainly necessary to perform an accurate correlation between the laboratory tests and PD-L1 values. Although our study has certain limitations such as its retrospective character and numerically incomparable research and control groups, it is so far the largest group among liver recipients that has been analyzed; it is also the only one that has enrolled children. Another important point is that we could evaluate specimens taken at different times after transplantation, as the biopsies included in the study covered many years of our work. We were also the first to use the division of PD-L1 expression in different cells, which appears to be crucial due to the different possible locations of PD-L1 in the liver.

## 5. Conclusions

Based on our findings, it seems that assessment of tissue PD-L1 expression may be a possible, new, and promising marker of acute cellular rejection and liver damage. Therefore, it is worth considering the determination of PD-L1 in liver biopsies, in “indication” biopsies as a further marker for ACR, and in protocol biopsies to identify patients with a higher risk for subsequent ACR. This association should be confirmed in further studies.

## Figures and Tables

**Table 1:** RAI score.

RAI Score	Number of Patients
2	1
3	6
4	4
5	4
6	3
8	1

**Table 2:** The grade of fibrosis in the protocol biopsies and ACR groups as assessed using Ishak scale.

Ishak Score	Protocol	ACR
0	12	3
1	10	2
2	14	4
3	5	2
4	8	2
5	4	2
6	2	0

**Table 3:** Clinical data.

	Protocol	ACR
Numer of patients	55	14 *
Age at transplantation (years)	0.12–17.9	0.53–16.5
	mean 5.13	Mean 4.44
	mead 2.97	Mead 1.99
Age at biopsy (years)	2.37–18.9	1.48–17.51
	Mean 12.74	Mean 9.49
	Mead 13.87	Mead 7.93
Diagnosis:		
Biliary atresia	32	5
Biliary cirrhosis other than BA	5	0
reLtx/rereLtx	4	4
AIH/PSC	2	1
ALF	3	2
Liver tumor	4	1
Other	5	1
Elective transplantation	33	6
Urgent transplantation	5	3
Acute on chronic	14	4
Oncological reason	3	1
Time from Tx to liver biopsy (years)	0.97–16.96	0.62–14.39
	Mean 7.62	Mean 3.52
	Median 8.36	Median 1.33

* ACR group included thirteen patients, but one patient had two transplantations and ACRs after each liver transplantation.

**Table 4:** Immunosuppression data.

	Protocol	ACR
	55	14 *
Primary immunosuppression		
Double drugs (CNI + steroids)	15	3
Double drugs (CNI + MMF)	32	7
Triple	8	4
Immunosuppression during biopsy		
Monotherapy (CNI or m-Tor inh)	32	3
Steroids monotherapy	1	2
Double (CNI + streroids)	5	3
Double (other)	13	1
Triple	3	5
Missing data	1	0
Actual immunosuppression		
Monotherapy (CNI or m-Tor inh)	25	0
Steroids monotherapy	1	0
Double (CNI/m-Tor inh + streroids)	15	6
Double (other)	8	0
Triple	6	8

* ACR group included thirteen patients, but one patient had two transplantations and ACRs after each liver transplantation.

**Table 5:** Biochemical parameters at time of liver biopsy.

	Protocol	ACR
	55	14 *
Total bilirubine (mg/dl)	0.13–36	0.21–11.8
Mean	2.56	2.37
Median	0.58	1.17
sGOT	9–79	20–346
Mean	31.38	117.26
Median	26	80
sGPT	5–117	15–569
Mean	27.89	170.05
Median	20	140
GGTP	0.41–509	10–1239
Mean	40.44	261.21
Median	23	183
INR	0.47–2.1	0.88–1.6
Mean	1.09	1.08
Median	1.08	1.06

* ACR group included thirteen patients, but one patient had two transplantations and ACRs after each liver transplantation.
